# Privacy-preserving method for face recognition based on homomorphic encryption

**DOI:** 10.1371/journal.pone.0314656

**Published:** 2025-02-11

**Authors:** Zhigang Song, Gong Wang, Wenqin Yang, Yunliang Li, Yinsheng Yu, Zeli Wang, Xianghan Zheng, Yang Yang

**Affiliations:** 1 The Academy of Digital China, Fuzhou, Fujian, China; 2 College of Computer and Data Science, Fuzhou University, Fuzhou, Fujian, China; 3 School of Business, East China University of Science and Technology, Shanghai, China; 4 School of Computing and Information Systems, Singapore Management University, Singapore; Jinan University, CHINA

## Abstract

In recent years, facial recognition technology has been widely adopted in modern society. However, the plaintext storage, computation, and transmission of facial data have posed significant risks of information leakage. To address this issue, this paper proposes a facial recognition framework based on approximate homomorphic encryption (HE_FaceNet), aimed at effectively mitigating privacy leaks during the facial recognition process. The framework first utilizes a pre-trained model to extract facial feature templates, which are then encrypted. The encrypted templates are matched using Euclidean distance, with the final recognition being performed after decryption. However, the time-consuming nature of homomorphic encryption calculations limits the practical applicability of the HE_FaceNet framework. To overcome this limitation, this paper introduces an optimization scheme based on clustering algorithms to accelerate the facial recognition process within the HE_FaceNet framework. By grouping similar faces into clusters through clustering analysis, the efficiency of searching encrypted feature values is significantly improved. Performance analysis indicates that the HE_FaceNet framework successfully protects facial data privacy while maintaining high recognition accuracy, and the optimization scheme demonstrates high accuracy and significant computational efficiency across facial datasets of varying sizes.

## 1. Introduction

Facial recognition is a convenient, non-forgettable, and durable method of identity authentication. It is widely used in various scenarios such as access control, mobile payments, and transportation. However, as facial data is highly sensitive and involves personal identity and appearance features, it raises significant privacy concerns. The current practice of processing facial recognition data in plaintext can lead to serious privacy breaches, such as identity theft and unauthorized use. The facial feature template database, which stores and manages facial feature data, is particularly vulnerable. Malicious administrators or attackers could potentially reconstruct the original facial images from the data, enabling them to impersonate others and commit illegal activities.

Homomorphic encryption allows for computations on encrypted data, enabling data processing without exposing the original information. Incorporating homomorphic encryption into the facial recognition process helps protect the privacy of facial data. However, this approach introduces significant latency in the recognition process, which can negatively impact the user experience. To address these privacy concerns and the latency issues associated with homomorphic encryption, this paper proposes an improved privacy protection method for facial recognition based on homomorphic encryption. The main contributions of this paper are summarized as follows:

(1) Facial Recognition Framework with Enhanced Security:

A novel framework, HE_FaceNet, leverages approximate homomorphic encryption to secure facial recognition processes on cloud servers. Framework Composition: Consists of two primary stages—identity registration and identity verification—where the cloud server facilitates both, acting as an intermediary. Security Mechanisms: Includes processes like feature template extraction, template encryption, ciphertext feature matching, and identity verification, protecting the facial feature templates from vulnerabilities.

(2) Optimized Search Scheme for Improved Efficiency:

To counteract the increased search times associated with traditional homomorphic encryption, a clustering-based optimization scheme is introduced. Interactive Ciphertext Computation: Implements an interactive process between local clients and cloud servers with dual systems for securing facial data and clustering information. Clustering on Encrypted Data: Groups similar encrypted facial data within clusters, streamlining the search by focusing on the closest cluster to a query, thus limiting the search area and reducing ciphertext matches. Enhanced Efficiency and Privacy: This optimization minimizes computational costs and improves recognition speed while safeguarding privacy, making it suitable for databases of varying sizes and different k-values.

(3) Framework Integration for Accuracy Testing:

HE_FaceNet integrates with the optimization scheme, rigorously tested for recognition accuracy and efficiency, confirming that privacy can be upheld without compromising recognition performance.

The remainder of this paper is structured as follows: Section 2 reviews related work in this field; Section 3 introduces the facial recognition framework based on approximate homomorphic encryption; Section 4 presents the optimization scheme for facial recognition based on clustering algorithms; Section 5 provides experimental tests of the HE_FaceNet framework and optimization scheme; and Section 6 concludes the paper.

## 2. Related work

Numerous studies have proposed improved methods for protecting privacy in facial recognition systems. Wang et al. [1] introduced a technique using discrete random standard orthogonal transformations to convert original biometric features into functions defined by user-specific keys. However, this approach struggles to handle significant variations within individual users’ data. Moujahdi et al. [2] employed random projection techniques on feature vectors to create irreversible transformations of biometric templates, resulting in non-reversible facial templates. However, this method requires that the transformation functions maintain both discriminability and irreversibility, which can be challenging to achieve. Boult et al. [3] combined the strengths of feature information transformation and encryption-based protection schemes, proposing a hybrid solution, though this approach may sometimes compromise recognition accuracy. Ross et al. [4] developed a scheme based on grayscale extended visual cryptography, encrypting a facial image into two separate images stored on different hosts. The original facial image can only be reconstructed when both images are available, but this method necessitates additional storage space for multiple shares. Guo et al. [5] used a local binary pattern approach, demonstrating good recognition rates under various lighting conditions, though the transmission of raw biometric data remains vulnerable to different types of attacks. Chamikara et al. [6] applied differential privacy techniques to perturb facial features, storing only the perturbed data on third-party servers to run standard feature recognition algorithms, but this can reduce recognition accuracy.

Some studies have incorporated homomorphic encryption into facial recognition systems. Erkin et al. [7] proposed a privacy-enhanced facial recognition system capable of concealing biometric templates while still achieving correct matching results. This method employs Paillier homomorphic encryption and provides an effective protocol for processing two sets of Paillier-encrypted data, although the integer-based additive homomorphic encryption used in this approach results in relatively low recognition accuracy. Liu et al. [8] introduced a chaotic encryption facial recognition algorithm based on Ridgelet-DCT transformation and the Tent-Henon map (THM). Unlike traditional methods, this approach first encrypts facial images using homomorphic encryption, extracts visually robust features, and then designs an encrypted facial recognition algorithm using a neural network model. Joshua et al. [9] proposed a method for conducting large-scale database searches within the encrypted domain, requiring both probe and gallery images to be represented in fixed-length formats. This encryption scheme is independent of how the fixed-length representation is obtained, making it applicable to any domain requiring fixed-length representations. Li et al. [10] combined the Paillier homomorphic encryption algorithm with an inner product protocol to construct a ciphertext-based facial recognition system. Nakanishi et al. [11] developed a system that enhances user accessibility by integrating multiple companies’ facial recognition engines, promoting the widespread application of facial recognition technology in societal structures. Jiang et al. [12] introduced a new approach by integrating partial homomorphic encryption into cloud-based facial identity verification, replacing Euclidean distance with Manhattan distance for enhanced security. Wang et al. [13] proposed a more efficient and secure PUM (Privacy preserving security Using Multi-key homomorphic encryption) mechanism for facial recognition. By integrating feature grouping with parallel computing, we enhance the efficiency of homomorphic operations. The use of multi-key encryption ensures the security of the facial recognition system. This approach improves the security and speed of facial recognition systems in cloud computing scenarios, increasing the original 128-bit security to a maximum of 1664-bit security. Bharat et al. [14] proposed a novel technique that combines Fully Homomorphic Encryption (FHE) with an existing template protection scheme known as PolyProtect. The embeddings can be compressed and encrypted using FHE and transformed into a secure PolyProtect template using polynomial transformation, for additional protection. Sun et al. [15] proposed a face feature ciphertext authentication scheme based on homomorphic encryption. Firstly, the face image feature extraction is completed based on the deep learning model. Secondly, the face features are packaged into ciphertext using homomorphic encryption and batch processing technology, and the face feature ciphertext is saved in the Cloud as a Service database. Thirdly, combined with automorphism mapping and Hamming distance, a face feature ciphertext recognition method is designed to complete the face feature ciphertext recognition in the case of ciphertext. Finally, the one-time MAC authentication method is used to ensure the integrity and consistency of the face feature ciphertext recognition results before and after decryption.Choi et al. [16] proposed Blind-Match, a novel biometric identification system that leverages homomorphic encryption (HE) for efficient and privacy-preserving 1:N matching. Blind-Match introduces a HE-optimized cosine similarity computation method, where the key idea is to divide the feature vector into smaller parts for processing rather than computing the entire vector at once. By optimizing the number of these parts, Blind-Match minimizes execution time while ensuring data privacy through HE.

## 3. Improvement framework for face recognition based on approximate homomorphic encryption

### 3.1. Overview

**Fig 1:**
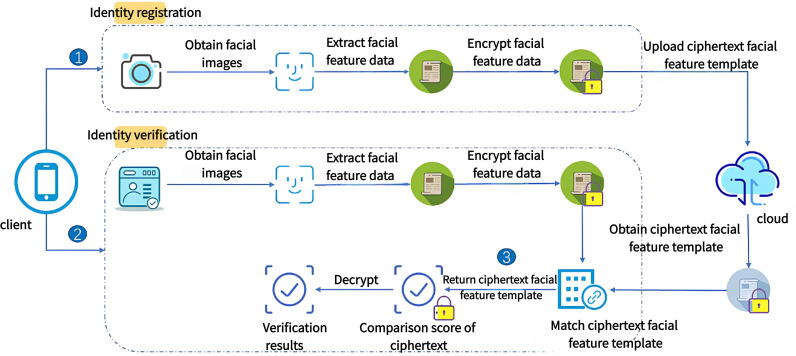
HE_FaceNet frame diagram.

The HE_FaceNet framework, which is based on approximate homomorphic encryption, is designed to protect user facial feature templates stored in cloud databases. By employing homomorphic encryption, the framework ensures that feature data is encrypted during the matching process, making it impossible for attackers to retrieve the actual facial images or corresponding feature attributes, even if the template database is compromised. The structure of the HE_FaceNet framework is illustrated in Fig 1.

The HE_FaceNet framework is primarily composed of two key components: identity registration and identity verification.

During the identity registration process, users upload facial images to the client through methods such as photography or video recording. The system employs deep learning algorithms to extract feature data from these images, which is then encrypted using homomorphic encryption. The encrypted facial feature templates are subsequently uploaded to the cloud. This process significantly reduces the risk of facial feature template leakage by avoiding the direct upload of plaintext facial feature data to the cloud.

In the identity verification process, the client first captures the user’s facial image that needs to be verified, extracts the facial feature data, and encrypts this data using a public key before uploading it to the cloud. The cloud then performs a homomorphically encrypted matching of the facial feature templates, utilizing the capability of homomorphic encryption to compute on encrypted data. The encrypted comparison score is sent back to the client. Finally, the client decrypts the received encrypted comparison score using a private key to obtain the final identity verification result.

### 3.2. HE_FaceNet framework method

#### 3.2.1. Feature template extraction.

**Fig 2:**
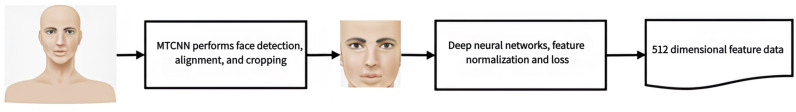
Schematic diagram of feature template extraction.

The HE_FaceNet framework utilizes a pre-trained FaceNet model to extract facial feature templates. This model is trained on the CASIA-WebFace dataset and employs the Inception ResNet v1 architecture. The process of facial detection and alignment is handled by MTCNN, which is responsible for detecting, aligning, and cropping the images. Afterward, the FaceNet model processes the images through a deep neural network, performing feature normalization to enable feature embedding. The entire feature extraction process is optimized using the triplet loss function, ensuring that the extracted facial feature data is represented in a 512-dimensional high-dimensional space. The detailed feature extraction process is illustrated in Fig 2.

#### 3.2.2. Feature template encryption.

To prevent malicious attacks on the facial feature template library, which could lead to the leakage of real facial images and their corresponding feature attributes, the extracted feature templates are immediately encrypted using approximate homomorphic encryption. Subsequently, the encrypted feature templates are securely transmitted to the cloud server. The specific process for generating keys and encrypting feature templates is outlined as follows:

(1) Key Generation Phase

The key generation function of the homomorphic encryption module is invoked to generate the necessary key components, including public key, private key, relinearization key, and rotation key. Once generated, the private key is securely stored on the client side. The public key, relinearization key, and rotation key are transmitted to the cloud. The cloud-stored keys are used for complex homomorphic computations, including noise control with the relinearization key for managing noise introduced by multiplication homomorphic operations. The rotation key is utilized during Euclidean distance computation to support more complex data operations.

(2) Feature Template Encryption

After extracting the feature templates, encryption processing is required. Encryption of the feature template vectors involves data type conversion, specifically converting the vector into a DoubleVector type. Once the conversion is completed, the vector data undergoes encryption processing to obtain the encrypted feature templates. Finally, the client uploads the encrypted feature templates to the feature template database.

#### 3.2.3. Cipher feature matching.

The framework employs a polynomial encryption method within approximate homomorphic encryption, meaning it can only perform approximate homomorphic operations on linear functions. For non-linear function models, approximate processing is necessary to transform them into approximate linear functions. The FaceNet model uses Euclidean distance between feature embeddings to compute similarity of feature templates. A smaller Euclidean distance indicates a higher likelihood that two feature templates represent the same individual. From the N-dimensional space Euclidean distance calculation formula, it is evident that all operations, except for the final square root computation, are linear.

In the cloud, after performing the $\overset{n}{\underset{i=1}{\sum }}{\left(x_{i}-y_{i}\right)}^{2}$ homomorphic calculation, the homomorphic result is returned to the client for square root calculation in plaintext. The homomorphic encryption module currently does not implement a direct summation of ${\left(x_{i}-y_{i}\right)}^{2}$. Therefore, vector rotation according to the respective dimensions is necessary to achieve summation of the first element. The specific implementation process of encrypted Euclidean distance algorithm is illustrated in Algorithm 1.

**Table:** 

Algorithm 1 Ciphertext Euclidean distance algorithm
Input: *cipher1* and *cipher2* Output: ciphertext squared and *cipher_sub_sq_sum*
*cipher*_sub ← *HE*. *sub*_*ciphertext* (*cipher1*, *cipher2*); //Calculate the difference between the two input ciphertext feature templates and store the result in cipher_sub. *cipher*_*sup*_*sq* ← *HE*. *square*_*ciphertext* (*cipher*_*sub*); //Square cipher_sub and store the result in cipher_sub_sq. *rotated* ← *Ciphertext* (*sipher*_*sub*_*sq*); //Rotate cipher_sub_sq and store it in rotated. *evaluator* ← *Evaluator* (*context*); //Create an evaluator for ciphertext evaluation. g*al*_*keys* ← *load*_key(*gal*_*keys*_path); // Load Galois keys. **FOR** *i* ← 1 ***TO*** *k* ***DO*** evaluator. rotate_vector_inplace(rotated, 1, gal_*keys*); //Rotate rotated by using the evaluator. *cipher*_*sub*_*sq*_*sum* ← *HE*. *add*_ciphertext(*rotated*, *cipher_sub*_*sq*); //Calculate the sum of rotated and cipher_sub_sq and store it in cipher_sub_sq_sum. **END FOR** **RETURN** *cipher*_*sub*_*sq*_*sum*.

#### 3.2.4. Face identity verification.

Upon receiving the transmitted encrypted comparison scores, the client decrypts them to obtain the plaintext sum of squares. To calculate the Euclidean distance between feature embeddings, the client performs a square root operation on the plaintext sum of squares. Based on this computation result, the client can verify face identity by comparing it against a predefined threshold. The specific implementation process is illustrated in Algorithm 2.

**Table:** 

Algorithm 2 Identity verification algorithm
Input: *cipher1* and *cipher2* Output: *ciphertext squared* and *cipher_sub_sq_sum*
*plain*_*score* ← *HE*. *decrypt*(*cipher*_*score*, *secret*_*keys*_*path*); // Decrypt the ciphertext comparison scores. *plain*_*score*_*sqrt* ← *math*. *sqrt*(*plain*_*score*); // Take the square root of the decrypted score. **IF** *plain*_*score*_*sqrt* ≤ *threshold* **THEN** ** RETURN** *TRUE*. **ELSE** ** RETURN** *FALSE*. **END IF**

### 3.3. Security analysis

In the assumed system model, all entities (including clients and the cloud) are honest but curious, meaning they can honestly execute the protocol’s computational processes but may attempt to obtain data from other entities during system operation. The adversary A * is defined to have the following capabilities:

1. A * may eavesdrop on the transmission of facial feature data.
2. A * may eavesdrop on intermediate results during facial feature template matching on the cloud, such as the squared Euclidean distance, and attempt to deduce other privacy data based on these intermediate results and its known facial data.

Regarding the protection of facial feature templates, clients upload encrypted data to the cloud after homomorphic encryption, with the private part of the key held exclusively by the client. Therefore, the cloud cannot decrypt the ciphertext feature templates, ensuring that the cloud cannot directly access users’ facial information. Intermediate results obtained through homomorphic operations (like squared Euclidean distances) also require the private key for decryption, preventing the cloud and attackers from recovering original data from ciphertexts. Thus, the focus is on the security of approximate homomorphic encryption itself, while the hardness assumption of learning with errors on rings ensures the security of approximate homomorphic encryption. The specific proof process is as follows:

1. If A * eavesdrops on the transmission of facial feature data, it will only obtain encrypted facial feature data. The security of approximate homomorphic encryption depends on the confidentiality of the private key, which only the entity holding the client’s private key can decrypt. Since A * cannot obtain the client’s private key, it cannot decrypt the facial feature template.
2. If A * eavesdrops on the cloud’s intermediate results during facial feature template matching, such as squared Euclidean distances, these results are generated through ciphertext addition and multiplication operations under the approximate homomorphic encryption framework, ensuring their security. Therefore, A * similarly cannot obtain any original information from these intermediate computation results.

## 4. Face recognition optimization scheme based on clustering algorithm

The HE_FaceNet framework leverages homomorphic encryption to enable the computation and storage of data under encryption. However, as the volume of data increases, the time required for ciphertext computation grows exponentially. To address this issue, this section introduces an optimized facial recognition scheme based on clustering algorithms, building upon the homomorphic encryption-based approach.

This optimization scheme first performs ciphertext clustering on the database, pre-structuring the facial ciphertexts stored in the database. Then, during the facial recognition process, the system utilizes the pre-defined clusters to perform ciphertext clustering-based facial recognition. This approach allows for more efficient retrieval and matching of the current facial data by narrowing down the search to specific clusters, thereby significantly enhancing the efficiency of large-scale encrypted feature value searches.

### 4.1. Improvement of clustering algorithm

**Fig 3:**
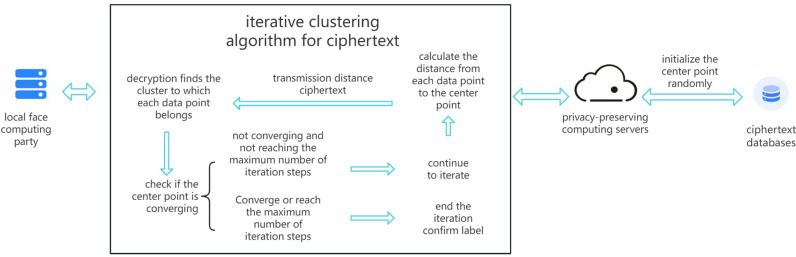
Flow chart of system ciphertext clustering.

The cloud server generates a new encryption key and uses it to encrypt pre-selected cluster center vector data. Locally, the encrypted template of facial features computes the Euclidean distance to the cluster centers, decrypts it to plaintext for comparison, ensuring all facial data points are closest to their respective cluster centers. The convergence of center points is checked; if not converged and maximum iteration steps are not reached, the cloud server generates new cluster centers. Through iterative processes, the convergence requirement is eventually met. Fig 3 illustrates the process of system ciphertext clustering.

### 4.2. Solution design

This solution primarily consists of two core steps: ciphertext clustering of the database and ciphertext clustering for face recognition. In the phase of ciphertext clustering for the database, the solution processes ciphertext data within the database to optimize data organization and reduce the computational burden of subsequent search steps. Next, in the step of ciphertext clustering for face recognition, distances of each cluster center are calculated first, followed by feature matching within the nearest cluster. Through this method, the solution not only maintains the accuracy of face recognition and data security but also significantly improves the efficiency of ciphertext computation matching.

**Fig 4:**
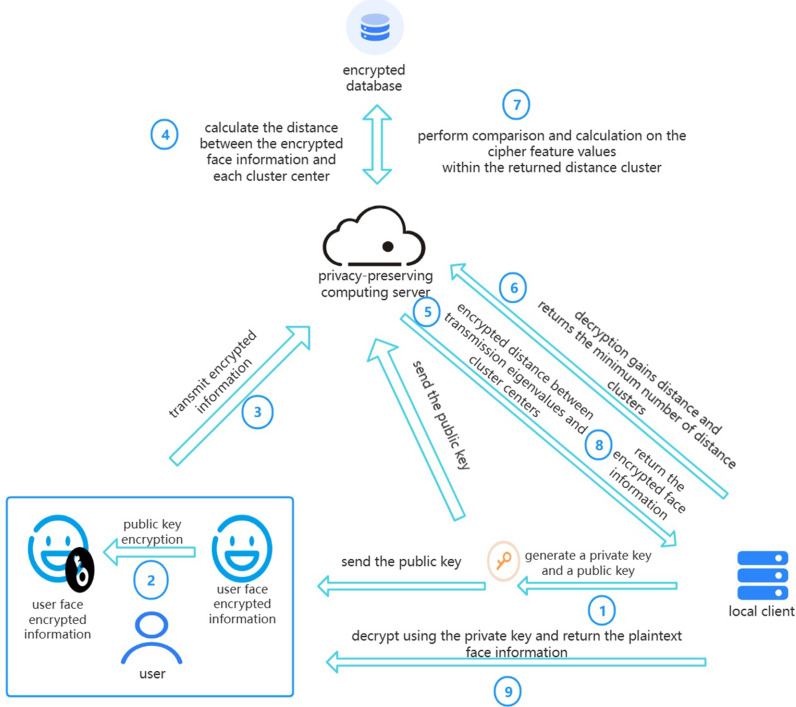
Flowchart of face recognition for system clustering.

The local client generates private and public keys, encrypts the public key along with facial information, and uploads it to the cloud server. The cloud server calculates encrypted facial feature ciphertexts using the ciphertext database and computes their Euclidean distances to each cluster center. After transmitting the encrypted Euclidean distance ciphertexts back to the local client, they are decrypted using the local private key. The local client then sorts the distances of clusters in ascending order and returns the sorted ciphertext indices to the cloud server. The server updates the dictionary by computing and comparing the Euclidean distances of each cluster iteratively until the minimum distance falls below a threshold. Fig 4 illustrates the above process.

#### 4.2.1. Encrypted database clustering.

(1) Initialization of Cipher Text Cluster Centers

Firstly, a suitable value for k needs to be chosen, which represents a crucial parameter for dividing the dataset into clusters. Subsequently, k random data points are selected as the centroids for clustering. These centroids will be used to compute the distance between each data point and its nearest cluster centroid, establishing the connection between each facial data point and the cluster centers. Following this, the cloud undertakes new key registration and generates keys, using these new keys to encrypt all cluster centroid vector data for subsequent computations.

(2) Calculation of Cipher Text and Cluster Center Distances

For each facial ciphertext feature value, it is necessary to calculate the ciphertext Euclidean distance from each cluster center. The formula for judging Euclidean distance in plaintext for facial feature values is as follows:


$$dist\left(X,Y\right)=\sqrt{\overset{i=1}{\underset{n}{\sum }}{\left(x_{i}-y_{i}\right)}^{2}}$$ (1)

Using the cipher subtraction function, we obtain the cipher difference in Euclidean distance:


$$\left(x_{i}-y_{i}\right)$$ (2)

Next, using cipher multiplication, we obtain:


$${\left(x_{i}-y_{i}\right)}^{2}$$ (3)

Finally, using cipher addition and cipher rotation, we calculate the cipher square root of the Euclidean distance between ciphertexts and cluster centers:


$$\sqrt{\overset{i=1}{\underset{n}{\sum }}{\left(x_{i}-y_{i}\right)}^{2}}$$ (4)

The cloud records the final computed cipher square root of the Euclidean distance. Simultaneously, the cloud encrypts the cluster centers and facial ciphertext numbers using the newly generated keys. These are then transmitted to the local area for the next decryption operation.

(3) Local Processing of Clustering Data

At this stage, using the cipher distances transmitted from the cloud, decryption is performed using locally stored private keys to obtain all feature distances between facial data points and cluster centers. In plaintext, comparisons are made between various characteristics and the nearest cluster centers, assigning each data point to the cluster of its closest center. This process effectively classifies data points. Subsequently, locally encrypted cluster information and encrypted facial ciphertext data are transmitted to the cloud for further computation to generate new cluster centers.

(4) Cloud Computation of New Cluster Centers

The cloud receives cluster information transmitted from local sources, decrypts using cloud keys, and obtains each cluster’s identifier along with encrypted facial data identifiers within each cluster. For each cluster, after successful data point classification, ciphertext computations yield the average ciphertext values of all data points within the cluster. These average values are then adopted as the new cluster centers. At this further computation stage, the cloud successfully acquires new cluster center information for each cluster from local transmissions, along with cluster assignments for each data point. For each cluster, the cloud computes the ciphertext Euclidean distance between the old and new cluster centers:


$$dist\left(K_{k}^{old},K_{k}^{new}\right)=\sqrt{\overset{i=1}{\underset{n}{\sum }}{\left(K_{ki}^{old}-K_{ki}^{old}\right)}^{2}}$$ (5)

Subsequently, the cloud transmits the Euclidean distances between old and new cluster centers for each cluster to local sources for further computation.

(5) Local Convergence Check

Locally, the cloud obtains distances between old and new cluster center vectors transmitted from the cloud, decrypting them using private keys. It evaluates these distances against the distances from the previous transmission of cluster center vectors. It computes the sum of distances $sun\left(dist\left(K_{k}^{old},K_{k}^{new}\right)\right)$ to determine if it is less than a threshold or if the maximum iteration count has been reached. If the sum of distances is not less than the threshold and the maximum iteration count has not been reached, it continues with the above steps. After each iteration, the iteration count increments until the sum of distances is less than the threshold or the maximum iteration count is reached.

Finally, the cloud records data identifiers contained within each cluster, encrypts their facial identifiers, and uses newly registered cloud keys to encrypt each cluster identifier. Encrypting cluster identifiers prevents access by entities other than the cloud while allowing the cloud to determine the cluster with the minimum distance in future computations.

#### 4.2.2. Encrypted clustering for face recognition.

(1) Calculating Cluster Membership from Cipher Text

The local client encrypts the current facial information X using a local key and transmits it to the cloud server. The server computes the encrypted Euclidean distance between the cipher text feature of the face and each cluster center:


$$dist\left(X,Center\right)=\sqrt{\overset{i=1}{\underset{n}{\sum }}{\left(x_{i}-center_{i}\right)}^{2}}$$ (6)

The calculated encrypted Euclidean distances are then transmitted back to the local client. Upon receiving the encrypted distances from the cloud, the local client decrypts them using its private key. It sorts and records the encrypted cluster indices based on distances from smallest to largest. The sorted encrypted cluster indices are then sent back to the cloud for further computation.

(2) Finding Corresponding Face within Clusters

Upon receiving the locally returned sorted encrypted cluster indices, the cloud server first decrypts the cluster index with the smallest distance. It sequentially computes the encrypted squared Euclidean distances between all cipher text features within that cluster and the current cluster center. It then transmits the encrypted person identifier along with the cipher text result back to the local client. The local client decrypts the received cipher text and person identifier using its private key, calculates the square root to obtain the final plaintext Euclidean distance:


$$dist\left(X,Y\right)=\sqrt{\overset{i=1}{\underset{n}{\sum }}{\left(x_{i}-y_{i}\right)}^{2}}$$ (7)

Using the obtained facial Euclidean distance, the dictionary is updated. The minimum distance is retrieved from the dictionary, and it is checked whether this distance is less than a predefined threshold derived from practical experience. If it is, the corresponding person identifier from the dictionary is returned; otherwise, a multi-retrieval strategy is initiated.

(3) Multi-Retrieval Strategy

If the facial distance obtained from the retrieval is greater than the threshold, a multi-retrieval strategy is required. Upon receiving the locally sorted cluster indices, the cloud server decrypts the smallest distance cluster index that has not been retrieved. It sequentially computes the encrypted squared Euclidean distances between all cipher text features within that cluster and the current cluster center, repeating the previous step. The server checks whether the retrieved squared Euclidean distance is less than the threshold. If it is, the corresponding person identifier from the data dictionary is returned; otherwise, the process is repeated. During multiple retrievals, the current smallest distance cluster is searched. Each time, a certain amount of retrieval time is added, significantly reducing the number of retrievals compared to a complete database traversal.

### 4.3. Time optimization analysis

Since the experiment involves clustering calculations with different values of k for varying sizes of facial databases, it is necessary to determine the k value that minimizes time consumption for different database sizes. Assuming clustering is performed on N facial images, divided into k clusters, and the time taken for encrypted vector matching of Euclidean distances is $T_{c}$, the total time consumption in a conventional computation process, which involves traversing and searching all facial feature values in the encrypted facial database, can be expressed as:


$$M_{0}=N\text{*}T_{c}$$ (8)

In this experiment, a ciphertext clustering retrieval scheme is used for fast retrieval of encrypted facial data. Initially, matching ciphertext distances with all cluster centers consumes $k\text{*}T_{c}$. Subsequently, retrieval within selected clusters is needed. With N faces divided into k clusters, although the number of faces allocated to each cluster may vary, on average, each cluster contains N/k faces. Therefore, the average time consumed for retrieval within each cluster is $N/k\text{*}T_{c}$. The total time consumption for the ciphertext clustering retrieval scheme is:


$$M_{cluster}=\left(\frac{N}{k}+k\right)\text{*}T_{c}$$ (9)

To achieve the minimum theoretical total time consumption $M_{cluster}$, we derive when $M_{cluster}$ attains its minimum value. Beginning with the equation:


$${\left(\sqrt{\frac{N}{k}}-\sqrt{k}\right)}^{2}\geq 0$$ (10)

Since the square of any value is greater than or equal to zero, expanding the square gives:


$$\frac{N}{k}+k-2\text{*}\sqrt{N}\geq 0$$ (11)

This simplifies to:


$$\frac{N}{k}+k\geq 2\text{*}\sqrt{N}$$ (12)

The left-hand side of the equation achieves its minimum value when $\frac{N}{k}=k$, or $k=\sqrt{N}$. Thus, the minimum total time consumption $M_{cluster}$ is achieved at:


$$M_{cluster}=\left(\frac{N}{k}+k\right)\text{*}T_{c}\geq 2\text{*}\sqrt{N}\text{*}T_{c}$$ (13)

Therefore, the theoretically most efficient k value is $\sqrt{N}$.

## 5. Performance analysis

### 5.1. Environment setup

The experiments were conducted in an Ubuntu virtual machine running on a server equipped with an Intel Core i7-11800H @ 2.30GHz CPU and 32GB of RAM. The LFW (Labeled Faces in the Wild) dataset was used for the experiments. This dataset includes 13,233 images of 5,749 individuals, with approximately 1,680 individuals having more than two facial images. The images vary in lighting, pose, expression, and resolution, simulating a wide range of real-world scenarios. Each image has dimensions of 250 millimeters in both width and height. While most images are in color, a small number are black-and-white. For subsequent facial recognition performance analysis, 3,000 matching and 3,000 non-matching pairs of faces were selected from the LFW dataset.

### 5.2. Accuracy analysis

#### 5.2.1. HE_FaceNet frame accuracy analysis.

**Fig 5:**
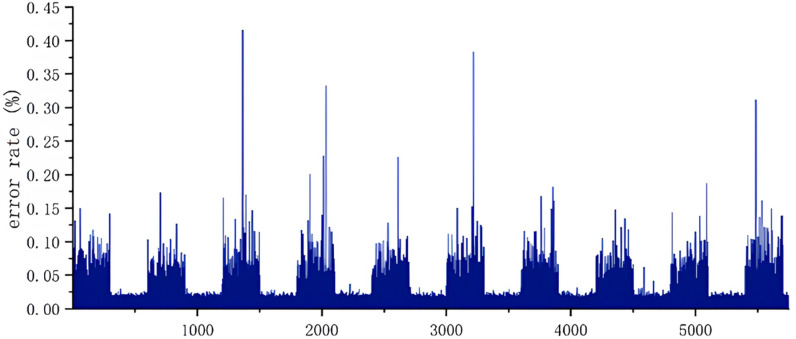
HE_FaceNet error rate.


Facial verification aims to determine whether two facial images belong to the same person, providing a basis for accuracy analysis of the HE_FaceNet framework. The error rate calculation formula for the HE_FaceNet model is shown in Equation 14. Fig 5 presents the verification results based on 6,000 face pairs, with an average error rate of 0.033206714%. This result indicates that the similarity calculations performed by the HE_FaceNet framework closely match the calculations done in plaintext.

$$errorrate=\frac{ABS\left(FaceNetScore-HE\_FaceNetScore\right)}{FaceNetScore}\times 100\text{\%}$$ (14)

where the numerator part calculates the absolute value of the difference between the similarity scores of two face images calculated by the FaceNet scheme and the HE_FaceNet scheme, and the denominator part is used to normalize the absolute deviation, so that the error rate can reflect the relative deviation degree with respect to the calculation result of the FaceNet scheme.

#### 5.2.2. Optimization scheme accuracy analysis.

Using the ciphertext clustering retrieval scheme for ciphertext search may impact the accuracy of facial recognition. There is a relationship between recognition accuracy and retrieval time; as the k-value reaches a turning point, the retrieval time is minimized, but the accuracy may also decrease. To address the potential decline in retrieval accuracy due to efforts to increase efficiency, the proposed scheme involves multiple searches within the current closest cluster. Since only the cluster with the current minimum distance from the cluster center needs to be searched, the time consumed is less than that required for searching the entire database, making multiple cluster searches feasible.

**Fig 6:**
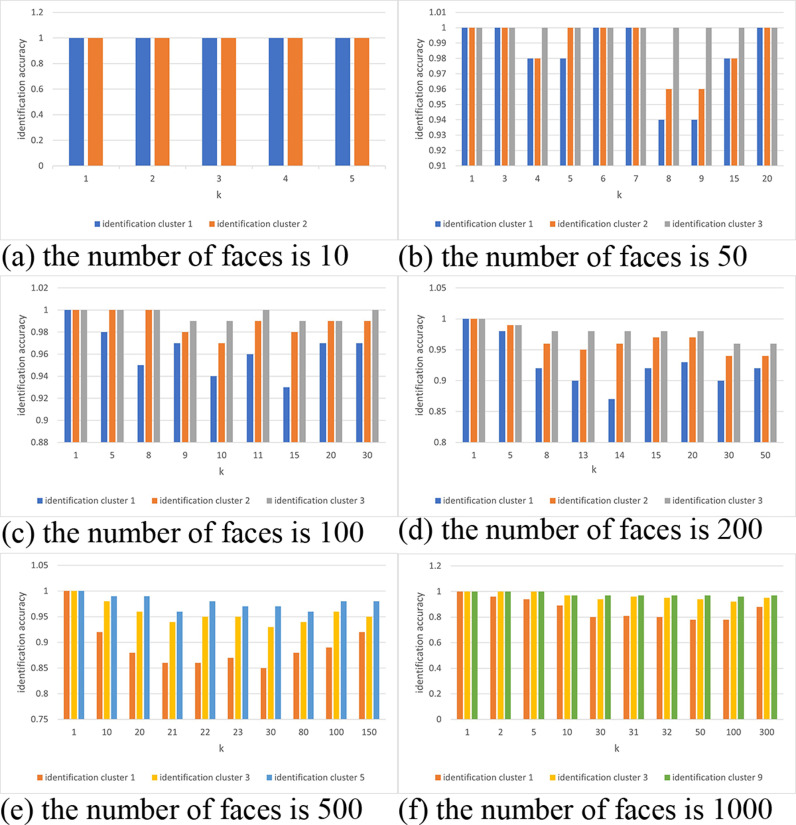
Accuracy Comparison of K-means Encryption Scheme. (a) the number of faces is 10. (b) the number of faces is 50. (c) the number of faces is 100. (d) the number of faces is 200. (e) the number of faces is 500. (f) the number of faces is 1000.

The face recognition accuracy under multiple cluster retrievals is shown in Fig 6. When the database contains 10 faces, each cluster achieves 100% accuracy. With 50 faces, the accuracy corresponding to the optimal k value in the second cluster reaches 96%. For 100 faces, the optimal k value in the third cluster retrieval achieves an accuracy of 97%. When the database contains 200 faces, the second cluster achieves 97% accuracy at k =  14. With 500 faces, the minimum accuracy of the fifth cluster at k =  21 reaches 96.4%. Finally, when the database contains 1,000 faces, the accuracy after multiple cluster retrievals reaches 97.2% at k =  30. This shows that the optimization scheme of using multiple cluster retrievals can maintain high accuracy across different scales of face databases while achieving efficient acceleration. The relationship between recognition accuracy and retrieval time is managed by searching only the cluster with the current minimum distance from the cluster center, reducing the search time without sacrificing accuracy.

### 5.3. Efficiency Analysis

#### 5.3.1. Efficiency analysis of the HE_FaceNet framework.

This section discusses the time consumption of the face verification scheme. The entire process mainly includes the following parts: feature template extraction, feature template encryption, ciphertext feature matching, and ciphertext feature decryption. The calculation results show that the average time for feature template extraction is 959.536 milliseconds, feature template encryption takes an average of 21.5 milliseconds, ciphertext feature matching takes 1832.35 milliseconds, and ciphertext feature decryption takes 2.002 milliseconds. For the calculation of 6000 pairs of samples, the HE_FaceNet scheme’s average time consumption increased by 1.855852 seconds compared to the FaceNet scheme. Compared with the FaceNet scheme, the primary additional time consumption of the HE_FaceNet scheme comes from feature template encryption, ciphertext feature matching, and ciphertext feature decryption, while the time consumption for feature template encryption and ciphertext feature decryption is relatively low. This is because most of the computation in the ciphertext feature matching phase is focused on the rotation and summation of ciphertext vectors. Although the increase in time is within an acceptable range, it brings significant advantages in privacy protection by encrypting and safeguarding sensitive facial data from exposure.

#### 5.3.2. Efficiency analysis of the optimization scheme.

In this scheme, the LFW dataset was first consolidated into datasets containing 10, 50, 100, 200, 500 and 1,000 facial images, respectively. Pre-encrypted data from these datasets were clustered, with the coordinates of each cluster center and the names within each cluster stored in ciphertext. For each of these databases containing different numbers of facial images, K-means clustering-based facial recognition was performed with varying k-values. The computation time for the K-means encrypted scheme was recorded, along with the speedup ratio compared to the standard encryption scheme. The speedup ratio refers to the improvement multiple of the optimized scheme (K - means encryption scheme) in computational efficiency compared to the ordinary encryption scheme. It is calculated by comparing the time consumed by the two schemes when processing the same task. The higher the speedup ratio, the more effective the optimized scheme is in improving the processing speed.

In the LFW dataset, two images were randomly selected for each individual: one for clustering in the database and the other for retrieval as a test image, forming the facial dataset and the face retrieval test set. Experiments constructed cluster centers with different k-values for datasets with varying amounts of facial data. The test set faces were used to perform facial retrieval within the cluster centers, returning the face with the smallest Euclidean distance in terms of feature values. If the test face and the returned face are two images of the same person, the retrieval is considered successful; otherwise, it is considered a failure.

**Fig 7:**
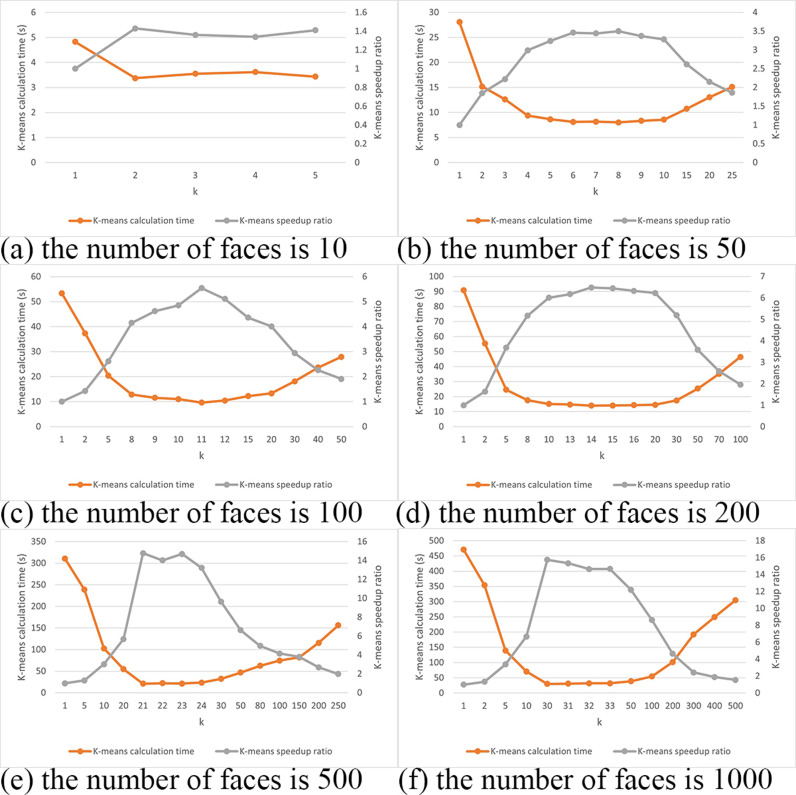
Efficiency Test of K-means Encryption Scheme. (a) the number of faces is 10. (b) the number of faces is 50. (c) the number of faces is 100. (d) the number of faces is 200. (e) the number of faces is 500. (f) the number of faces is 1000.

The efficiency test results of the K-means encryption scheme are shown in Fig 7. With a database of 10 faces, the highest theoretical acceleration ratio is achieved at k =  3. In the experimental setup, the acceleration ratio at k =  3 is 1.36, while the maximum acceleration ratio of 1.43 occurs at k =  2. For 50 faces, the highest theoretical acceleration ratio is at k =  7; experimentally, k =  7 yields an acceleration ratio of 3.44, with the maximum acceleration ratio of 3.50 at k =  8. For 100 faces, the theoretical maximum acceleration ratio is at k =  10; however, in the experimental environment, the maximum ratio of 5.54 is reached at k =  11. With 200 faces, the theoretical maximum is at k =  14, and the experimental setup achieves this at k =  14 with an acceleration ratio of 6.49. For a database of 500 faces, the theoretical maximum acceleration ratio is at k =  22, but experimentally it is reached at k =  21 with a ratio of 14.77. Finally, for 1,000 faces, the theoretical maximum is at k =  31, while the experimental setup achieves the highest ratio of 15.75 at k =  30.These results demonstrate that the proposed scheme significantly improves processing speed across various scales of facial databases, with particularly notable acceleration in large-scale datasets.

### 5.4. Comparative analysis

#### 5.4.1. Comparative analysis of the HE_FaceNet framework.

**Fig 8:**
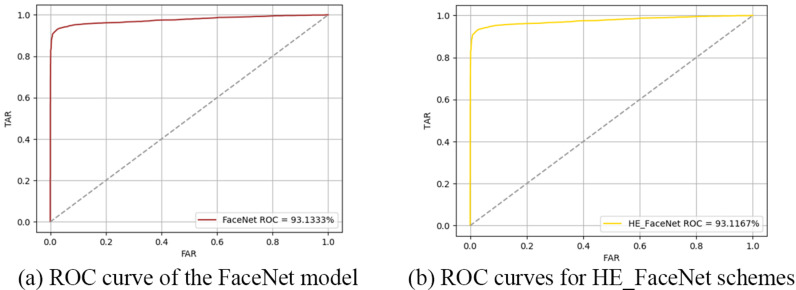
ROC curve comparison of FaceNet model with HE_FaceNet ramework. (a) ROC curve of the FaceNet model. (b) ROC curves for HE_FaceNet schemes.

**Fig 9:**
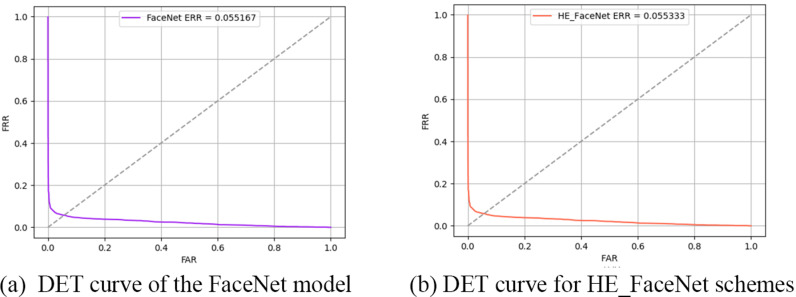
DET curve comparison of FaceNet model with HE_FaceNet ramework. (a) DET curve of the FaceNet model. (b) DET curves for HE_FaceNet.

Further experiments compared the FaceNet model with the HE_FaceNet framework, with the results shown in Figures 8 and 9. The ROC (Receiver Operating Characteristic) value for FaceNet is 93.1333%, while that for HE_FaceNet is 93.1167%. This indicates that the homomorphic computations in HE_FaceNet have a minimal impact on recognition accuracy, which is consistent with the findings from the error analysis.

In biometric recognition systems, the goal is to minimize both the False Accept Rate (FAR) and the True Accept Rate (TAR), although there is often a trade-off between these two metrics. When one improves, the other might deteriorate. The Equal Error Rate (EER) of the FaceNet and HE_FaceNet models is presented through the Detection Error Tradeoff (DET) curve, which plots FAR against the False Rejection Rate (FRR). The EER for FaceNet is 0.055167, while for HE_FaceNet, it is 0.055333. A DET curve closer to the origin indicates better system performance. These results demonstrate that the approximate homomorphic calculations in HE_FaceNet have a negligible effect on the system’s EER.

#### 5.4.2. Comparative analysis of the optimization scheme.

The proposed scheme uses ciphertext data for clustering, whereas the method in reference [17] performs clustering on plaintext data. In this scheme, ciphertext is transmitted and computed between the cloud and the local client during the clustering process. The clustering is performed in the ciphertext domain, allowing the cloud server to cluster the data without accessing plaintext information, thereby preventing facial information leakage either in the cloud server or during transmission. Consequently, the security of this scheme is superior to those where plaintext clustering is performed in the cloud, as shown in other studies. A comparison of facial recognition accuracy between this scheme and those from related literature is presented in Table 1.

**Table 1:** Accuracy comparison of ciphertext recognition schemes.

Scheme	Identification Rate	Number of clusters selected k
Ref. [17] Single match	0.933	15
Ref. [17] Multiple Match	0.979	15
Ref. [7]	0.964	—
This Scheme	0.984	20

Additionally, since multiple searches do not involve traversing all faces but rather increase the search time for a single cluster, the overall impact on retrieval time is minimal. For example, for a database with 1,000 faces, where the minimum retrieval accuracy corresponds to 200 clusters, the experiment only consumed 1/20th of the time required for full-cluster retrieval. Therefore, the time consumed by multi-cluster retrieval is relatively small compared to the entire retrieval process.

## 6 Conclusion

This paper proposes a privacy protection method for facial recognition based on homomorphic encryption, addressing the current shortcomings in privacy protection within facial recognition technologies. By incorporating approximate homomorphic encryption, an improved facial recognition framework, HE_FaceNet, was designed to enable effective ciphertext matching after the encryption of facial feature templates, thereby ensuring the security of users’ facial data during transmission and storage. Additionally, to address the issue of slow efficiency in homomorphic encryption solutions, an optimization scheme based on clustering algorithms was introduced, significantly enhancing computational efficiency in large-scale facial databases. This scheme clusters similar faces together, reducing the range and time required for ciphertext searches. Experimental results demonstrate that the HE_FaceNet framework effectively protects facial data privacy while maintaining recognition accuracy. Moreover, the optimization scheme notably improves computational efficiency across different scales of facial datasets, particularly in large-scale scenarios. In summary, the proposed method not only offers significant advantages in privacy protection but also excels in both accuracy and efficiency. This research provides robust technical support for real-world applications of facial recognition systems, with potential for widespread use in areas such as access control, payments, and transportation.
